# Mortality from Cholera Influenced by Elevation

**Published:** 1854-02

**Authors:** 


Mortality from Cholera influenced by Elevation.—It was discovered during the epidemic cholera of 1848–9, in London, that the rate of mortality by that disease was nearly in the inverse proportion of the elevation of the ground on which the dwellings of the inhabitants stood. The same relation between the rates of mortality at different elevations, though the deaths have been comparatively few, has hitherto been observed in the present epidemic. The mortality from Cholera in the districts at an average elevation of less than 20 feet above Trinity high-water mark has been 31 in 100,000 inhabitants; in the districts of an average elevation of 20 and below 40 feet (20—40 feet) the mortality has been 16 in 100,000; at an elevation of 40—60 feet the mortality has been 11 in 100,000; at 60—80, it has been only 4; at 80—100, only 3. Marylebone, at an average elevation of 100 feet, is the only exception to the law; the mortality has been there 13 in 100,000. At Hampstead, where the elevation may be put at 350 feet, there has hitherto been no death from Cholera. Exceptional circumstances disturb the average in particular districts; but it is a general rule that the danger of dying of cholera, and of all plagues, diminishes within certain limits in proportion as the dwellings of the population are raised above the level of the sea. —*Med. Times and Gazette.*

